# More than a co-incidence? Comment on: Amyotrophic lateral sclerosis is over‐represented in two Huntington’s disease brain bank cohorts: further evidence to support genetic pleiotropy of pathogenic HTT gene expansion

**DOI:** 10.1007/s00401-022-02517-1

**Published:** 2022-11-06

**Authors:** Hannah S. Bakels, Stephanie Feleus, Vera van Dis, Susanne T. de Bot

**Affiliations:** 1 Department of Neurology, Leiden University Medical Center, Leiden, The Netherlands; 2 Department of Epidemiology, Leiden University Medical Center, Leiden, The Netherlands; 3 Department of Pathology, Leiden University Medical Center, Leiden, The Netherlands; 4 Department of Pathology, Erasmus University Medical Center, Rotterdam, The Netherlands

With interest we took notice of the recent paper by Hickman and colleagues on the over-representation of Amyotrophic Lateral Sclerosis (ALS) pathology in Huntington Disease (HD) brains [4]. The current study appears as a sequel on earlier reported pathogenic *HTT* gene expansions (PHGE) in ALS patients, suggesting a causal pathogenetic link between *HTT* gene expansions and ALS pathology [3]. Hickman and colleagues evaluated ALS pathology in two HD brain bank cohorts and revealed an overlap of ALS and HD pathology in 0.8% of cases, as compared to none in an Essential Tremor (ET) cohort and a one year prevalence of 0.0052% of ALS in the general population of the United States of America [5]. In turn, this finding would support their hypothesis of genetic pleiotropy in PHGE patients.

Although the current study reveals interesting findings in this respect, shortage of comprehensive methodological information limits the interpretability and reproducibility of these findings. Most importantly, limited information is provided on the cohort baseline characteristics, assessment methods and used diagnostic criteria. Information on age of the material, disease stage, age at death and other potential confounding factors is not available for the full cohorts. This could for example mask a potential survival bias if donors in the essential tremor (ET) cohort are older than those in the HD brain bank. Those who developed ALS while alive, are less likely to be included in the ET brain bank. Simply because they would then classify as ALS patient and would not survive long enough to develop (severe) ET. Introduction of a survival bias could explain why no ALS pathology was found in the ET cohort.

A second limitation arises with the described overlapping HD/ALS cases. The inclusion of HD brains in this study appears to be based on neuropathological evidence (Vonsattel grade 1 or higher), even in the absence of clinical (case #1, #4 and #6) or genetic evidence of HD (case #4 and #6). The choice not to include clinical and genetic criteria for HD into the dataset, increases the risk of confounding results. For instance, overlapping neuropathological hallmarks for HD and ALS, such as neuronal loss in the motor cortex, brain stem nuclei and striatum, as well as p62 and TDP43 inclusion bodies and or translocations, are all described before [1, 6–8]. Together with the minimal methodological information that is provided, makes it questionable if neuropathological findings in these cases can be attributed to one disease process or the other. In addition, one could argue that case #1 has been mistakenly entered in an HD brain bank, since an HD phenotype in life was questionable in this patient (and only based on expanded HTT carriership), therefore causing a potential misclassification bias.

Something else to consider is the inequal comparison that is introduced, by comparing ALS *neuropathology* in HD brains with the prevalence of a *clinical* ALS phenotype in the general US population. Pathology of neurodegenerative disease is known to precede a clinical diagnosis for decades. The true number of cases with ALS *neuropathology* (without yet a clinical diagnosis) in the general population is therefore unknown and likely to be higher (though probably not by 100-fold). The authors seem to resolve this inequal comparison using the comparison with ET brains, serving as ‘general population’. However, this introduces another risk of bias, next to the potential survival bias already mentioned. One could argue that reassessment of the *initial* neuropathology reports, of both HD and ET brains, gives a higher a priori likelihood of finding ALS neuropathology in HD brains than in ET brains, based on their clinical overlap and common neurodegenerative pathomechanisms. This introduces an information bias since the primary neuropathological assessment methods are in all probability not equal for the HD and ET cohorts. For example, neuropathology assessment methods often differ between pathology departments. It is unclear which brain regions were assessed and which immunohistochemical stainings were performed in each brain, including the ET cohort. In case the use of protein aggregation markers differs between the HD and ET cohorts, the a priori chance of finding ALS pathology in one or the other cohort may differ. Another uncertainty is caused by the used reference of Brownell et al., as it does not clarify standardized criteria for histopathological ALS findings [2]. Standardized, blinded microscopic reassessment of all brains would help to eliminate this bias. In addition, comparison with another neurodegenerative cohort (i.e. Alzheimer’s Disease, Parkinson’s Disease, Lewy Body Dementia) would, in our opinion, be informative as well.

Nevertheless, highlighting overlapping genetic, pathological and clinical features in neurodegenerative disease is important and helps us to understand common pathomechanisms between phenotypes. More extensive information on material and methods in combination with the attribution of risks and confounders would greatly help in appreciating the presented findings. Also, cautiousness in the way findings are presented in the title and discussion part would greatly help in this matter. Amyotrophic lateral sclerosis neuropathology was found in two Huntington’s disease brain bank cohorts; more than a co-incidence?
